# Correlates of Urinary Tract Infections Among Women of Reproductive Age in India: A Systematic Review

**DOI:** 10.7759/cureus.58681

**Published:** 2024-04-21

**Authors:** Aarushi Mavi, Isha Rathi, Mohd Shannawaz, Shazina Saeed, Shamimul Hasan

**Affiliations:** 1 Epidemiology and Public Health, Amity Institute of Public Health and Hospital Administration, Noida, IND; 2 Biostatistics and Epidemiology, Amity Institute of Public Health and Hospital Administration, Noida, IND; 3 Oral Medicine and Radiology, Faculty of Dentistry, Jamia Millia Islamia, New Delhi, IND

**Keywords:** risk factors, india, pregnancy, women’s health, urinary tract infections

## Abstract

Urinary tract infections (UTIs) are a significant health concern globally, with a pronounced impact on women's health in India. This systematic literature review aims to elucidate the factors associated with UTIs among women of reproductive age in India and focus on demographic, behavioral, and physiological factors to inform targeted public health and clinical interventions.

A systematic literature search was conducted on PubMed and Google Scholar using specific MeSH terms and preferred reporting items for systematic literature reviews and meta-analyses (PRISMA) guidelines to investigate the correlates of UTIs among Indian women. Studies were selected based on their relevance to the correlates of UTIs among Indian women, including risk factors, prevention strategies, and treatment outcomes.

The review identified a significant prevalence of UTIs among pregnant women, with *Escherichia coli* being the most common causative agent. Younger women, particularly those pregnant, were found to be at a higher risk, likely due to physiological changes during pregnancy and increased sexual activity. Behavioral and lifestyle factors, such as inadequate water intake and poor sanitation practices, were strongly associated with increased risks for UTIs. Factors that increase the risk of UTIs in women include frequent sexual activity, involvement with a new sexual partner, spermicide use that can potentially alter vaginal pH and impact its bacterial composition, and vulvovaginal atrophy. Additionally, nearly 60% of women globally with recurrent UTIs experienced sexual dysfunction, indicating the broader implications of UTIs on women's sexual health and quality of life.

UTIs among women in India are influenced by a complex interplay of factors. There is a critical need for enhanced public health initiatives focusing on sanitation, hydration, and hygiene, alongside holistic clinical management strategies that address both the infection and its broader health impacts. Future research should aim at developing innovative prevention and treatment strategies, with a particular focus on high-risk groups such as pregnant women, to mitigate the burden of UTIs in India.

## Introduction and background

Urinary tract infections (UTIs) are among the most prevalent infections affecting women globally, with a significant impact observed during pregnancy due to accompanying anatomical and physiological changes. In India, the incidence of UTIs in women, particularly pregnant women, varies widely, indicating the influence of diverse biological, socio-economic, and lifestyle factors. This variability underscores the importance of identifying the specific correlates of UTIs to enhance preventive strategies and treatment modalities [1]. Research has highlighted a prevalence range for UTIs in Indian women from 3.14% to 19.87%, indicating the significant burden of this condition [2]. Factors such as parity, history of abortion, sexual behavior, water intake, and urination habits have been identified as key contributors to the increased risk of UTIs [3,4]. Given the complexity and multifactorial nature of UTIs, especially in rural settings, there is a pressing need for a comprehensive analysis to understand the dynamics influencing the prevalence of UTIs among Indian women [1-4].

Pregnancy causes several physical, hormonal, and functional changes in the urinary tract. This increases urine stasis and the ascending of microbially contaminated urine from the bladder into the ureters, causing UTIs. The changes in the urinary tract and immunological changes associated with pregnancy, along with an already short urethra, predispose women to UTIs [5]. Urinary tract infection (UTI) is one of the more common perinatal complications, affecting approximately 8% of pregnancies. These infections represent a spectrum, from asymptomatic bacteriuria (ASB) to symptomatic acute cystitis to the most serious, pyelonephritis [6]. The original criterion for diagnosing asymptomatic bacteriuria was more than 100,000 bacteria/mL on two consecutive clean catch samples. The detection of more than 100,000 bacteria/mL in a single voided midstream urine is accepted as an adequate and more practical alternative, although there is only an 80% probability the woman has true bacteriuria, increasing to 95% if two or more consecutive cultures are positive for the same organism. The global prevalence of ASB in pregnant women ranges from 2% to 15%, thus underscoring the importance of prompt screening and intervention [7,8]. In India, the lack of standardized screening protocols for ASB in pregnant women results in underestimations of its importance and adds to the variation in the reported prevalence rates [9-11].

There is a paucity of literature available on the burden of UTI in rural areas and primary and secondary care settings. This, along with the nonavailability of a facility for microbiological diagnosis at a subdistrict hospital, evoked our interest in estimating the burden of UTI and the feasibility of microbiological diagnosis of UTI at a subdistrict hospital.

This systematic review aims to investigate the correlates of UTIs among women of reproductive age in India, with a particular focus on pregnant women. The study intends to elucidate the various risk factors associated with UTIs, ranging from physiological changes during pregnancy to lifestyle and hygiene practices. Identifying these correlates will not only shed light on the prevalence of UTIs among Indian women but also inform targeted public health interventions and healthcare policies to mitigate the burden of this condition.

## Review

Materials and methods

A systematic review of the literature was conducted to evaluate the correlates of UTIs among women in India. This review adheres to the PRISMA (preferred reporting items for systematic reviews and meta-analysis) guidelines for the systematic review process [12].

Research Question

The PICO framework (population, intervention, control, and outcomes) was employed to outline the search strategy:

Population: Women of reproductive age (aged 15-49 years)

Intervention/exposure: UTIs

Control: Was not applicable in this study

Outcome: Correlates and contributing factors to UTIs in women.

This study aims to answer the following research question: “What are the factors contributing to UTIs among women of reproductive age in the rural and urban areas of India?”

Literature Search

The systematic review followed the preferred reporting items for systematic reviews and meta-analyses (PRISMA) 2020 guidelines [12]. We conducted a comprehensive literature search in the PubMed and Scopus databases, covering the period from January 2013 to December 2023. The search strategy was designed to capture studies evaluating the correlates, risk factors, and treatment outcomes of UTIs in women of reproductive age within the Indian context. The following Medical Subject Headings (MeSH) terms and their combinations were used to ensure a thorough search: "Urinary Tract Infections"[MeSH] OR "Urinary Tract Infections/epidemiology"[MeSH] OR "Urinary Tract Infections/therapy"[MeSH] OR "Urinary Tract Infections/prevention and control"[MeSH] OR "Urinary Tract Infections/complications"[MeSH] AND "Women's Health"[MeSH] OR "Women"[MeSH] AND "India"[MeSH] OR "India/epidemiology"[MeSH]. This search strategy aimed to identify studies that provided insights into the prevalence, risk factors, prevention strategies, and management practices for UTIs among women in India, thereby contributing to a better understanding and addressing this significant health concern. The following inclusion and exclusion criteria were considered (Table 1).

**Table 1:** Inclusion and exclusion criteria.

Inclusion criteria	Exclusion criteria
Studies conducted in India.	Excluded all the international studies.
Studies that assessed the prevalence and correlates of urinary tract infections among women.	Excluded females not in the age group of 15-49 years.
Included women participants in their reproductive age 15-49 years.	Articles published in languages other than English and before January 2013.
Full-text articles published in the English language between January 2013 and December 2023.	Not accessible in full text.

Study Selection

The titles and abstracts of the identified studies were assessed by two authors, and any disparity was resolved by a third author. Studies not evaluating the correlates of UTIs among women in India were excluded. However, when the study abstract lacked clarity, the complete texts were obtained for assessment and independently analyzed by two authors.

Data Extraction and Analysis

Data extraction was carried out to compile relevant findings regarding the factors associated with UTIs among women in India. The data extraction was done with Rayyan software and included information on publication metrics, including the first author's name, publication year, study design, study population, and identified causes and risk factors for UTIs. Due to the heterogeneity of the included studies, it was not possible to conduct a statistical evaluation of the results, and therefore, a meta-analysis was not performed.

Studies varied significantly in their methodological approaches, including cross-sectional, cohort, and case-control designs, which influenced how data were collected, analyzed, and reported. This variation underscored the complexity of comparing quantitative outcomes directly across studies. Instead, a qualitative synthesis of findings was conducted. This approach allowed for the identification of common themes and risk factors associated with UTIs in the selected demographic, notwithstanding the methodological differences. Special attention was given to the context of each study, considering the socio-economic and cultural diversity within India that could influence UTI risk factors and prevalence.

Risk of Bias-Assessment

Bias assessment was systematically carried out using the STROBE (strengthening the reporting of observational studies in epidemiology) checklist [13]. The STROBE checklist is a widely recognized tool designed to enhance the quality of reporting in observational studies, including case-control, cohort, and cross-sectional studies. The checklist comprises items that emphasize the importance of transparent reporting across several domains of study design and execution, such as study objectives, methods, results, and discussion.

Utilizing the STROBE checklist for risk of bias assessment in the study involves a detailed examination of the included studies based on these criteria. Each included study was evaluated for compliance with STROBE criteria, focusing on aspects such as the clear definition of study objectives, adequacy of participant selection, description of methods, presentation of results, and discussion of findings, including limitations. This assessment facilitated the identification of potential biases within and across studies, such as selection bias, reporting bias, and confounding factors, which could influence the interpretation of the results. The analysis was further informed by discussions on the implications of the identified biases for the overall findings of the review.

Results

Literature Research

**Figure 1:**
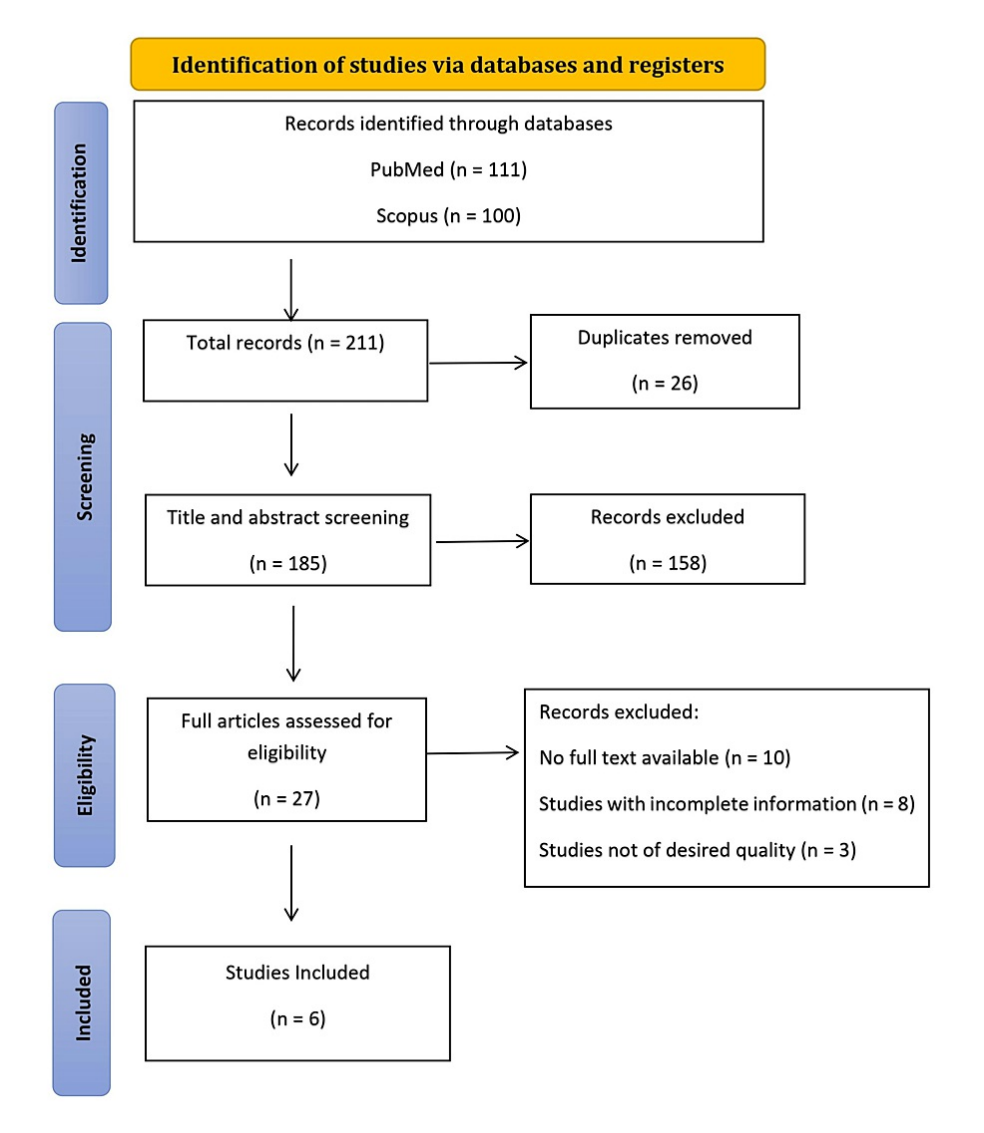
PRISMA flowchart PRISMA: Preferred reporting items for systematic literature reviews and meta-analyses

The initial search across electronic databases yielded a total of 211 articles. After removing duplicates, 185 unique records were screened based on their titles and abstracts. Upon reviewing the titles, 158 overlapping studies were excluded according to the exclusion criteria, leaving 27 records for further evaluation. The abstracts of these 27 articles were then reviewed. These 27 articles underwent a detailed assessment based on the inclusion and exclusion criteria. During this evaluation, 10 articles were excluded due to the unavailability of full text. Ultimately, 17 articles met the quality evaluation stage and were further assessed for their relevance to the study objectives. Among these articles, eight studies were excluded due to incomplete information, and three were excluded because the articles were not of the desired quality, resulting in a final selection of six studies for inclusion in the study. Figure 1 illustrates the selection process.

Study Characteristics

A summary of the information extracted from the reviewed articles is given in Table 2 [14-19].

**Table 2:** Detailed characteristics of the included studies.

Author (s)/Year	Type of Study	Study population	Findings	Causes of UTI
Philip PS et al. 2013 [14]	Cross-sectional survey	260 women aged 15-44 years	17.3% of urban married women aged 15-44 in Ludhiana reported symptoms of RTIs/STIs, with the most common being dysuria (57.8%) and frequent urination (53.3%). Significant risk factors included older age, higher gravidity, higher education, living in joint families, a history of abortion, and using ordinary cloth during menstruation. Among those with symptoms, 64.4% were untreated, and 68.7% of those treated only reported partial relief.	Having more pregnancies, higher education levels, living in joint families, having a history of abortion, and using ordinary cloth during menstruation.
Thakre SS et al. 2015 [15]	Observational study	300 pregnant women	9.6% prevalence of UTI among pregnant women, with *Escherichia coli* being the most common causative agent. Significant associations were found between UTI and several factors: multigravidity, water intake of less than one liter per day, the habit of postponing urination, and not voiding after intercourse.	Lack of clean water and improper personal hygiene.
Almeida-Velasco et al. 2019 [16]	Cross sectional study	577,768 women aged 15-49 years	25% of urban and only 4.3% of rural women used improved menstrual hygiene (MHM) methods, with cloth being predominantly used. Improved MHM was associated with fewer reproductive tract infections (RTIs), highlighting the need for better MHM practices to prevent RTIs, especially in rural areas.	Poor menstrual hygiene practices.
Vyas S et al. 2015 [17]	Cohort study	177 nursing students aged 18-30 years	The overall prevalence of UTI was found to be 19.8% (35/177). There is an urgent need to sensitize the nursing students regarding the growing need for the issue so that they themselves become aware, in addition to raising the awareness of other high-risk groups.	Rural background, inadequate water intake, and unsatisfactory toilet habits were found to be strong predictors of UTI.
Kant S et al. 2017 [18]	Case-control study	1253 pregnant women	33.3% of women reported symptoms suggestive of UTI, but only 3.3% were confirmed to have UTI through urine culture. A significant association was observed between the presence of UTI symptoms and the actual UTI diagnosis (OR = 7.35).	NA
Mondal S et al. 2022 [19]	Cross-sectional study	697 women of reproductive age	20.5% (143 women) had recurrent UTIs. Among these, 59.4% exhibited signs of sexual dysfunction based on the Female Sexual Function Index, and 58.1% reported sexual distress as indicated by the Female Sexual Distress Scale, significantly higher than those without recurrent UTIs.	Recurrent infections, possibly due to sexual distress or hygiene issues.

The six studies published between 2013 and 2023 constituted the final data set for the literature review and provided valuable insights into the correlates of UTIs among women of reproductive age in rural and urban areas of India. The selected articles contributed to the overall understanding of UTIs in this specific population. Among the six studies included, which covered a total of 580,455 females, three were cross-sectional studies [14,16,19], and one each was an observational study [15], a cohort study [17], and a case-control study [18], respectively.

Assessment of Risk of Bias

The risk of bias assessment for the selected studies was carried out using the STROBE checklist [13]. This evaluation aimed to determine the methodological quality and potential biases within each study to ensure the reliability and validity of our findings. The risk of bias assessment indicates that the majority of selected studies exhibit a "low to moderate" risk of bias [14,15,17-19], with one study having a "low" risk of bias rating [16]. This suggests that while there is generally a good level of methodological rigor and reporting clarity among the studies, the studies provide valuable insights into the correlates of UTIs among women in India, contributing to a nuanced understanding of this significant health issue, albeit with a degree of caution due to the potential biases identified in Table 3 [14-19].

**Table 3:** Risk of bias assessment following the STROBE checklist for selected studies.

Study Reference	Title and Abstract	Introduction	Method	Result	Discussion	Overall Risk of Bias
Philip PS et al. (2013) [14]	Yes	Yes	Partial	Yes	Yes	Low to moderate
Thakre SS et al. 2015 [15]	Yes	Yes	Partial	Yes	Partial	Low to moderate
Almeida-Velasco et al. 2019 [16]	Yes	Yes	Yes	Yes	Yes	Low
Vyas S et al. 2015 [17]	Yes	Yes	Partial	Yes	Yes	Low to moderate
Kant S et al. 2017 [18]	Yes	Yes	Partial	Yes	Partial	Low to moderate
Mondal S et al. 2022 [19]	Yes	Yes	Partial	Yes	Yes	Low to moderate

Study Outcome

The study highlights the complexity and variability of UTI prevalence among women in India, showcasing a prevalence range from 9.6% in pregnant women residing in rural areas [15] to 19.8% among nursing students, underscoring the influence of demographic and environmental contexts on UTI risks [17]. Noteworthy is the identification of several risk factors strongly associated with UTIs, such as multigravida status, inadequate hydration-specifically, consuming less than one liter of water per day-and reduced frequency of urination-not exceeding six times daily [15]. Furthermore, sexual behavior, particularly the frequency of intercourse exceeding three times per week and post-coital hygiene practices, emerged as significant predictors of UTIs, with *Escherichia coli* being the predominant causative agent in approximately 62.06% of UTI cases among pregnant women [14,18].

The consequential effects of UTIs extend beyond physical health, significantly impacting women's sexual function. Women of reproductive age experiencing recurrent UTIs reported lower female sexual function index (FSFI) scores, indicative of sexual dysfunction, in comparison to those without recurrent UTIs (59.4% versus 22.6%, respectively). This association is further corroborated by elevated female sexual distress scale (FSDS) scores, suggesting a profound impact on sexual distress [19]. Additionally, specific predictors of symptomatic UTIs identified among nursing students, including rural background, insufficient water intake, and inadequate toilet habits, emphasize the critical role of environmental and lifestyle factors [17]. Collectively, these studies underline the multifaceted nature of UTI risk factors among Indian women, incorporating elements of personal hygiene, sexual activity, lifestyle choices, and environmental conditions. The significant influence of recurrent UTIs on sexual health and functionality accentuates the necessity for holistic healthcare strategies that address both prevention and therapeutic interventions.

Discussion

This systematic review meticulously compiles and examines the diverse range of studies focusing on the correlates of UTIs among women in India, thus offering a nuanced perspective on the multifaceted risk factors that influence UTI prevalence. Specifically, the reported 9.6% prevalence of UTIs among pregnant women in Nagpur, predominantly attributed to *Escherichia coli*, echoes the global consensus that highlights *E. coli* as the principal pathogen in UTIs, suggesting that microbial factors play a significant role in the infection landscape within the country [15]. This statistic not only aligns with international data but also sheds light on the critical need for targeted microbial management strategies within the Indian healthcare context. Further demographic analysis reveals a notable concentration of UTIs among pregnant women, with a mean age of 23.17 years, suggesting that younger women, particularly those undergoing pregnancy, are at an elevated risk [20]. This demographic-specific vulnerability could be attributed to the complex interplay between increased sexual activity and the physiological transformations associated with pregnancy, which may predispose younger women to higher UTI risks [21]. Such insights underscore the importance of incorporating age and pregnancy status into UTI risk assessments and tailored prevention strategies.

Behavioral and lifestyle factors, including but not limited to inadequate water intake, infrequent urination, and certain sexual behaviors, have been identified as significant contributors to UTIs [14,16]. Holding urine for a long time has proven to be an important risk factor, and among the different reasons for holding urine, holding due to the poor sanitary condition of public toilets was the most common. The higher frequency of self-reported UTIs is related to the holding of urine, behavioral features, and attitudes of women [22]. Notably, the use of Western toilets and exposure to unsanitary public facilities were also significantly associated with increased UTI prevalence [15]. These findings highlight the indispensable role of personal hygiene and sanitation in mitigating UTI risks, advocating for enhanced public health campaigns and infrastructure improvements that promote clean and accessible sanitation facilities. Moreover, the impact of recurrent UTIs on sexual function, where nearly 60% of affected women experienced sexual dysfunction, illuminates the profound implications of UTIs beyond the immediate physical discomfort. This association with sexual dysfunction underscores the broader repercussions on women's sexual health and overall quality of life, amplifying the call for holistic clinical management strategies that address both the infection and its secondary impacts [19].

The synthesis of these findings points to an urgent need for multifaceted public health initiatives and clinical interventions that prioritize sanitation, hydration, hygiene, and awareness of sexual health practices [22]. Such initiatives are critical in not only preventing UTIs but also in mitigating their broader impacts on women's health and well-being. Furthermore, the complex and interrelated nature of UTI correlations calls for ongoing research to unravel the intricacies of UTI risks and develop targeted interventions. Future studies should aim to explore innovative prevention and treatment strategies, including microbial management, public sanitation improvements, and community-based health education programs, to effectively reduce the UTI burden among women in India.

## Conclusions

UTIs present a significant public health concern, particularly among pregnant women. Our review identifies a multifactorial risk landscape, encompassing microbial pathogens, demographic factors, behavioral practices, and lifestyle choices, with *Escherichia coli* emerging as the predominant causative agent of UTIs in the studied population. The demographic analysis underlines the heightened vulnerability of younger women, especially those who are pregnant, to UTIs. This suggests a critical window for targeted interventions and educational programs focused on this subgroup. Behavioral and lifestyle factors such as inadequate water intake, infrequent urination, and specific sexual behaviors significantly contribute to the risk of UTIs, emphasizing the importance of comprehensive health education and improved sanitation facilities. Moreover, the association of recurrent UTIs with sexual dysfunction highlights the profound impact of UTIs on women's overall health and quality of life. This underlines the necessity for holistic management strategies that not only aim at treating the infection but also address its broader implications for sexual health. Our findings call for enhanced public health initiatives that improve sanitation, promote proper hydration, and advocate for better hygiene practices among women.

There is a pressing need for tailored clinical management that incorporates prevention, early detection, and treatment of UTIs, especially among high-risk groups such as pregnant women. Future research should continue to explore the complex interplay of factors contributing to UTIs among women in India, focusing on innovative and culturally appropriate prevention and treatment strategies. Collaborative efforts between healthcare providers, policymakers, and communities are essential to effectively reduce the burden of UTIs, improve women's health outcomes, and enhance their quality of life. Thus, addressing the correlates of UTIs among women in India requires a multifaceted approach that includes public health interventions, clinical management, and ongoing research. By understanding and targeting the diverse risk factors associated with UTIs, we can make significant strides toward mitigating this pervasive health issue and ensuring a healthier future for women across India.
